# Pulsatile flow through idealized renal tubules: Fluid-structure interaction and dynamic pathologies

**DOI:** 10.3934/mbe.2020094

**Published:** 2019-12-17

**Authors:** Niksa Praljak, Shawn D. Ryan, Andrew Resnick

**Affiliations:** 1 Department of Mathematics and Statistics, Cleveland State University, Cleveland OH 44115, USA; 2 Department of Physics, Cleveland State University, Cleveland OH 44115, USA; 3 Center for Gene Regulation in Health and Disease, Cleveland State University, Cleveland OH 44115, USA

**Keywords:** fluid-structure interactions, modeling, pulsatile flow, kidney, physiology

## Abstract

Kidney tubules are lined with flow-sensing structures, yet information about the flow itself is not easily obtained. We aim to generate a multiscale biomechanical model for analyzing fluid flow and fluid-structure interactions within an elastic kidney tubule when the driving pressure is pulsatile. We developed a two-dimensional macroscopic mathematical model of a single fluid-filled tubule corresponding to a distal nephron segment and determined both flow dynamics and wall strains over a range of driving frequencies and wall compliances using finite-element analysis. The results presented here demonstrate good agreement with available analytical solutions and form a foundation for future inclusion of elastohydrodynamic coupling by neighboring tubules. Overall, we are interested in exploring the idea of *dynamic pathology* to better understand the progression of chronic kidney diseases such as Polycystic Kidney Disease.

## 1. Introduction

Time-dependent flow in liquid-filled elastic tubes is a longstanding object of research with broad applicability [1–3]; yet there is still much to be understood. In biology, a major application is flow within organ systems (e.g., cardiovascular, liver and kidney). One major barrier to kidney flow research is the lack of ability to perform *in vivo* flow measurements through a living renal tubule. For a brief review of the literature see [4]. The so-called *internal biofluiddynamics* [5] has been heavily studied by a large community of scientists and engineers [6, 7]; however there remains a need to develop a multiscale mathematical model to better predict and understand the relationship between applied fluid stresses and resultant tissue mechanotransduction mechanisms. Fluid flow has been shown to have significant biological implications regarding salt and water homeostasis [8, 9] and tubular structure itself [10–13]. Importantly, a cellular structure, the primary cilium, is hypothesized to function as a flow sensor [14,15] and cilia have been implicated in a variety of physiological and pathophysicological conditions [4]. Indeed, we are interested in the relationship between fluid flow stimulation of primary cilia and regulation of transepithelial salt and water transport.

While the study of internal biofluiddynamics can be traced back to Galen and Harvey [16, 17], the quantitative study of pulsatile viscous flow within elastic tubes originates with Womersley [18–20]. This work has been extended to include semi-permeable tubes [21–23] which can be used to model fluid and solute reabsorption, important metrics that parameterize kidney function.

**Figure 1.**
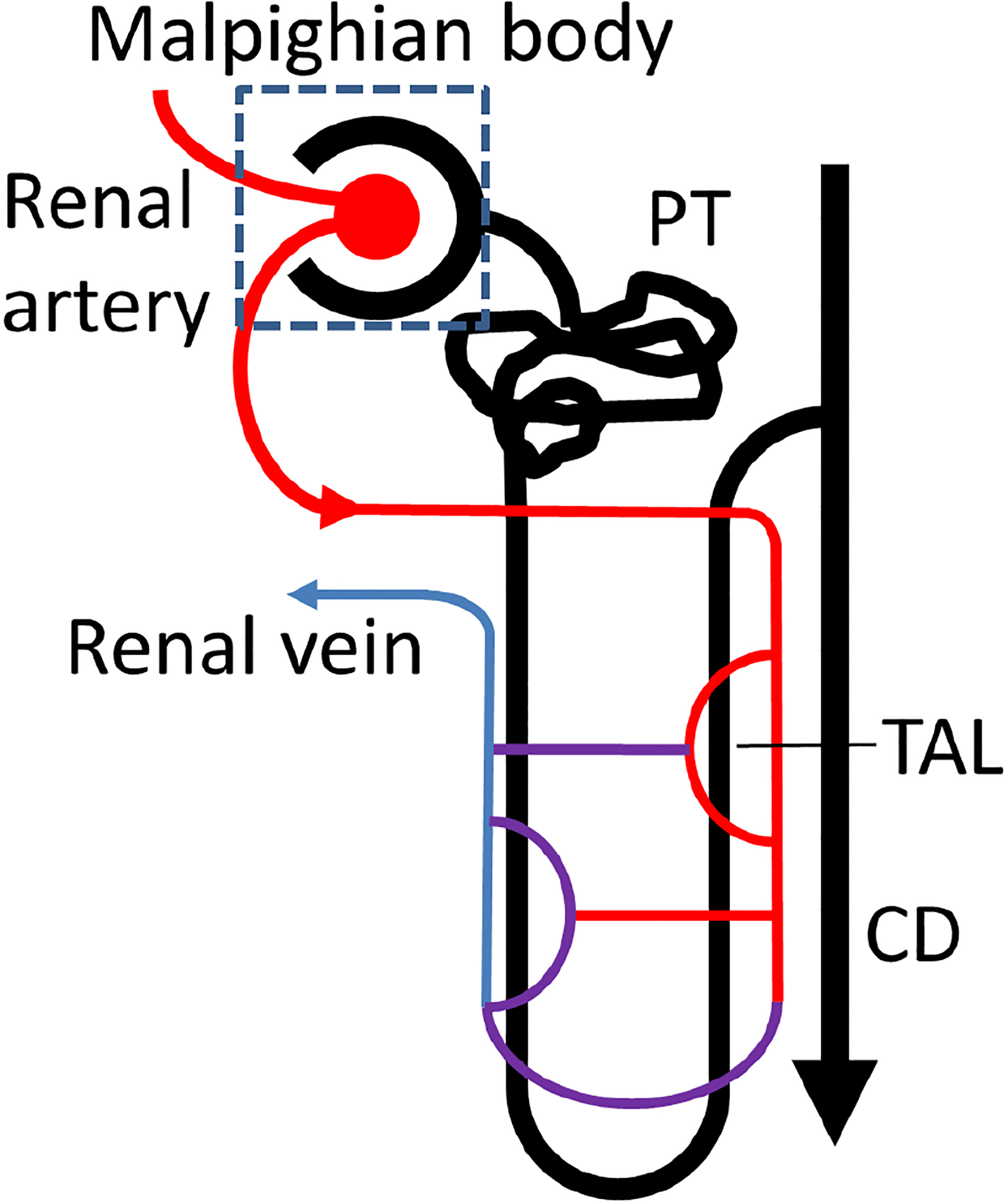
The nephron is the functional unit of the mammalian kidney, composed of a malpighian body and renal tubule. The malpighian body, located in the renal cortex, consists of a capillary tuft (glomerulus) that delivers blood from the cardiovascular system and is surrounded by a double-walled capsule, Bowman’s capsule. The glomerular filter separates blood and capsular space. Primary urine flows through the Proximal Tubule (**PT**) into the Loop of Henle which consists of a descending limb and thick ascending limb (**TAL**). The ultrafiltrate then moves through the distal tubule and empties via the collecting duct (**CD**) which opens into the renal pelvis, ureter and bladder. Salt and water balance is achieved by the selective transfer of fluid and solutes from the tubule to the renal vein.

As considered here (Figure 1), kidney anatomy consists of approximately *1 × 10*^*6*^ independent non-branching nephrons, each beginning with a malpighian body through which blood plasma enters via the glomerular filter and ending with the collecting tubule, which empties into the collecting duct, renal pelvis, and ureter. Nephron walls are single-cell thick, and as the fluid ultrafiltrate moves through a nephron, most > *99*% of the fluid is reabsorbed back into the blood. Current *in silico* models of renal flow exist for the proximal tubule [24, 25], and some *in silico* models exist for the Loop of Henle [26–28] and collecting duct [29]. Our work presented here is a crucial step in modeling the dynamics of interior renal flow. In this study, we focus only on accurately modeling the distal nephron (specifically, the cortical collecting duct) rather than the entire nephron. Broadly speaking, our ‘future work’ includes modeling the entire nephron by 1) incorporation of unsteady pressure perturbations, 2) incorporation of solute and water reabsorption and 3) to remove simplified cylindrical geometry assumptions. The effects of renal flow on primary cilia can be performed within the laboratory under well-controlled conditions [30,31], but the field suffers from a lack of knowledge about what fluid flow conditions are actually present, especially deep within the medulla.

A survey of available literature reveals a few salient facts regarding time-dependent fluid flow within a nephron. In [32], clear evidence of pulsatile flow within a living rat proximal tubule is presented via live cell microscopy of an oscillating primary cilium. The oscillation frequency, *4.5 Hz*, is correlated with the heart rate of anesthetized rats, and furthermore, proximal tubule pulsatile flow ceased at death. In [33], a mathematical model of the thick ascending limb demonstrated that compliant (elastic) tubule walls act to intensify harmonics; the driving frequency chosen was *30 mHz* and the output flow rate contained harmonics at *60* and *90 mHz*.

Two recent reports provide additional relevant information. In [34], a model of a nephron tree consisting of *22* interacting nephrons and connecting vasculature demonstrated both the synchronization of tubular pressure oscillations as well as both quasiperiodic and chaotic pressure perturbations within a single component nephron. In [35], a detailed computational model of a single complete nephron was developed, providing predictions of Glomerular Filtration Rate (GFR) and volume flow rate that are in reasonable agreement with experimental measurements.

Here, we investigate the dynamic behavior of an elastic cylindrical tube filled with an incompressible Newtonian fluid driven by pulsatile pressure variations using a macroscopic multiscale mathematical model. Solutions of the governing differential equations were obtained numerically, using COMSOL Multiphysics^®^ [36] finite-element analysis packages (e.g., Fluid-Structure Interaction). Results presented here examine the effect of varying the driving frequency on the flow over a range of wall compliances. Throughout this report, we assume the elastic tube to be impermeable. This approximation is most valid for the distal nephron (collecting tubule and collecting duct), segments that are responsible for the final “fine tuning” of salt and water resabsorption, approximately *0.5*%.

Furthermore, since we aimed to interrogate distal nephron dynamics, an adequate assumption in modeling the renal tubular structure is non-porous walls. However, within future studies focused in modeling excretion and reabsorption at the Loop of Henle, porous wall incorporation becomes imperative. Porous walls within cylindrical tubules are comparatively different to non-porous walls. In particular through a dispersion coefficient study, earlier work found that solute transport due to flow of a non-Newtonian Ostwald-de Waele fluid can be perturbed by porous walls compared to non-porous walls [37]. Also, the Loop of Henle creates an area of high urea concentration through implementations of electrolyte pumps, specifically producing an efficient process to reabsorb water and create a concentrated urine for excretion. Early work [38] presented an analytical model for transport of a chemical species within a cylindrical tube coupled to a porous medium, potentially creating a feasible basis for future modeling of the Loop of Henle.

**Figure 2.**
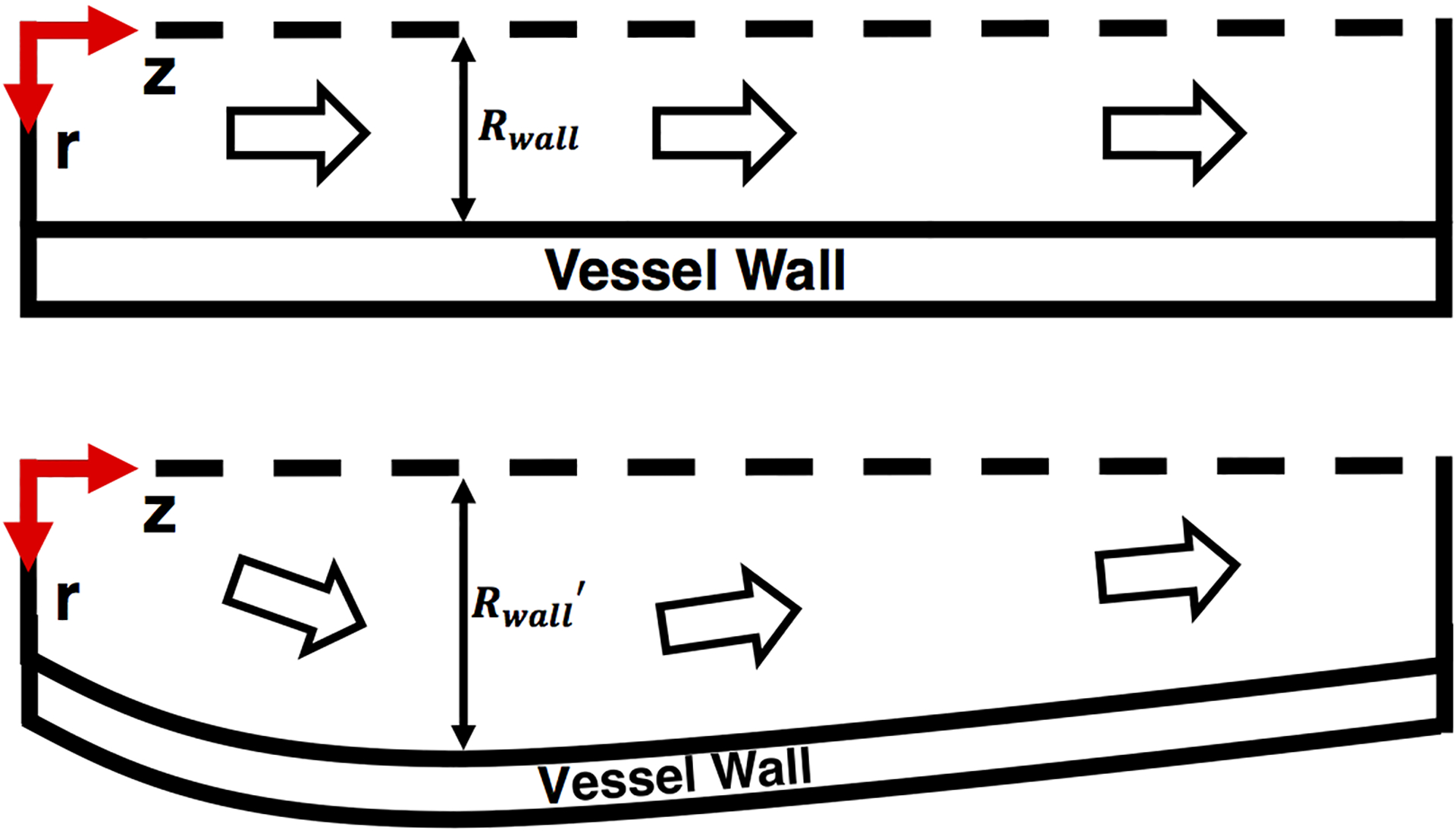
Schematic plan of axisymmetric geometry with fluid-wall interplay.

One of the aims of the work presented here is to investigate the fluid-structure interaction within a renal tubule (Figure 2) and identify possible flow regimes of interest: Resonant interactions, for example. One particularly intriguing concept is *dynamic pathology* [39], where the source of disease is not genetic, but rather a consequence of altered system dynamics due to chronic accumulated changes in system parameters (e.g., wall compliance).

This manuscript is ordered as follows. First, the development of the fluid-structure equations that model the distal nephron is presented. Secondly, the numerical computation and validation is analyzed and verified by investigating grid stability and solution accuracy. Then, the results from the numerical simulation are presented. Finally, our conclusions are discussed.

## 2. Mathematical model

We modeled a fluid-filled renal tubule as a combination of a homogeneous, incompressible, Newtonian fluid (with viscosity ‘*μ*’) and a circular cylindrical isotropic elastic tube with radius ‘*r*’. This simple model can be readily extended to incorporate transmural flow corresponding to salt and water reabsorption by semi-permeable tube walls; tubule-tubule interactions via the extracellular viscoelastic medium [40, 41]; and orthotropic modeling of the tube wall [42]. Flow is laminar and generated via the pulse, so we use the time-dependent Navier-Stokes momentum equations for the fluid, an elastic solid model for the tube wall, and analyze fluid-structure interaction via a FSI module in COMSOL^®^.

One important simplification is that the flow velocity does not have an azimuthal component; a second important simplification considered here is that bulk fluid properties (velocity ‘***u****(****t****)*’, density ‘*ρ*_*f*_ ‘, pressure ‘*p(t)*’) vary continuously in space and time. However, we must separately consider the fluid velocity and density at fluid-solid interfaces. This is due to the fact that the stress tensor is related to the velocity gradient, and the velocity must not have discontinuities at the fluid-solid interface (no-slip boundary condition [43]). We expect that the no-slip boundary condition must be relaxed when, for example, our model incorporates wall permeability [44]. Similarly, we expect that axisymmetry may be broken if there are neighboring tubules that can mechanically interact via the interstitium.

### 2.1. Fluid and structural equations

Understanding the temporal evolution of the fluid-wall interplay requires use of governing equations that characterize the dynamics of the renal tubule. Using parameter values relevant to renal physiology, we wish to model pulsatile flow of an incompressible viscous Newtonian fluid within an axisymmetric tube with varying pulsation frequencies and structural elasticities. Futhermore, our reference configuration is illustrated on the top row in Figure 2 and the coordinate system implement for modeling is cylindrical coordinates (*z*, *r*, *θ*). The fluid viscosity and density are denoted by *μ*_*f*_ and *ρ*_*f*_ . The fluid flow in the renal tubule is described by the incompressible Navier-Stokes equations for the velocity field ***u*** = (*u*_*z*_, *u*_*r*_) in the spatial (deformed) moving coordinate system:

(2.1) $$\rho_{f}\frac{\partial u}{\partial t}-\nabla \cdot \left[-pI+\mu_{f}\left(\nabla u+{\left(\nabla u\right)}^{T}\right)\right]+\rho_{f}\left(\left(u-u_{m}\right)\cdot \nabla \right)u=F,\;-\nabla \cdot u=0$$

In the above equations ***I***, ***F*** and ***u***_*m*_ = (*u*_*z*_,*u*_*r*_)_*m*_ represents the diagonal matrix, external volume force affecting the fluid, and the coordinate system velocity; in particular, we are assuming that no external forces are acting on our single renal tubule system – hence ***F*** = *0*. In future studies, we plan to incorporate neighboring tubules since nephrons are closely compacted in a mammalian kidney – thus ***F*** ≠ *0*.

We will define ***Ξ*** = ***Ξ****(z,r)* as the original location of a material particle. The variable ***Ξ*** represents the coordinate system of the solid structure throughout the deformation history; in other words, the coordinate system is known as the material frame. As the particle moves at a certain time *t*, we can represent the particle’s position with the spatial coordinate system (i.e., spatial frame) as ***ξ*** = ***ξ***(*Ξ*,*t*). By definition, ***Ξ*** is fixed to the body, while ***ξ*** is fixed in space. The displacement vector is *v*(*Ξ*,*t*); therefore, we can use the displacement vector as a transformation between the material to the spatial frame by ***ξ*** = ***Ξ*** + *v*(***Ξ***,*t*). This expression only holds true for local changes in the shape of the material. The deformation gradient ***Γ*** becomes

(2.2) $$\Gamma =\frac{\partial \xi }{\partial \Xi }=I+\frac{\partial v}{\partial \Xi }$$

where ***Γ*** represents a Jacobian matrix of the transformation between the material and spatial frame. Thus, the determinant of the deformation gradient is *X* = det (***Γ***). In addition, determinant of the deformation gradient can be expressed as the volume factor $X=\frac{dV}{dV_{o}}$ which provides valuable info into the volume change during deformations.

For an arbitrary undeformed material volume *V*_*o*_, the differential momentum balance equation for the solid body is

(2.3) $$\rho_{o}\frac{\partial^{2}v}{\partial t^{2}}=f_{V}+\nabla_{\Xi }\cdot P^{T}$$

where ***f***_***V***_ and ***P*** represents force per deformed volume (i.e., volumetric force) and first Piola-Kirchhoff stress tensor. Futhermore, we will introduce several known first Piola-Kirchhoff known relations:

(2.4) $$P=X\sigma \Gamma^{-T}=\tau \Gamma^{-T}$$

(2.5) $$P=\Gamma S=\frac{\partial \xi }{\partial \Xi }S=(I+\nabla v)S.$$

In the above equations, ***σ*** and ***τ*** represents the Cauchy stress tensor and the Kirchhoff stress tensor. In Eq (2.4), we have a relation between stress tensors, while Eq (2.5) expresses a direct relation between the first and second Piola-Kirchoff stress tensor; therefore, we can finally express Eq (2.3) with a momentum balance equation description for a linear elastic material with the constitutive relation ***S*** = ***S***(**Λ**) by substituting Eq (2.5) into Eq (2.3):

(2.6) $$\rho_{o}\frac{\partial^{2}v}{\partial t^{2}}=f_{V}+\nabla_{\Xi }\cdot (I+\nabla v)S(\Lambda ).$$

The variable ***Λ*** is the Green-Lagrange strain tensor where $\Lambda_{ij}=\frac{1}{2}\left(\partial_{\Xi_{j}}v_{i}+\partial_{\Xi_{i}}v_{j}+\partial_{\Xi_{j}}v_{k}\partial_{\Xi_{i}}v_{k}\right)$ in Einstein notation and $\partial_{\Xi }\equiv \frac{\partial }{\partial \Xi }$ for convenience. In addition, we can always change between perspectives by rewriting the displacement vector as ***Ξ***(***ξ***,*t*) = ***ξ*** − *v*(***ξ***,*t*). Equation (2.6) is important as it governs the linear elastic kinematics for the tubule wall, and the relation gives a closed system of equations for the displacement vector. For fluid-structure interplay, COMSOL handles the interactions using Arbitrary Lagrangian-Eulerian (ALE) formulation.

The ALE formulation involves using two different frames (e.g., Eulerian and Lagrangian) to describe the system. In our case, the solid domain will be formulated through the Lagrangian approach, while the fluid domain will be governed by an Eulerian framework. The fluid interacts with a tubule wall through pressure and viscous forces along the interacting boundary. In particular, the renal tubules are relatively compliant, so the load from the fluid causes a deformation which can lead to altered flow patterns. For this interplay, the constitutive relations between the fluid domain and wall domain at the interface are:

(2.7) $$u=\frac{\partial v}{\partial t}$$

(2.8) $${\left(\sigma \cdot n\right)}_{\text{fluid}}={\left(\sigma \cdot n\right)}_{\text{wall}}.$$

The above equations demands for equating between fluid-wall velocities and stresses at the interacting boundary through the fluid velocity, displacement vector, Cauchy stress tensor, and the normal vector.

### 2.2. Fluid and structural boundary conditions

**Figure 3.**
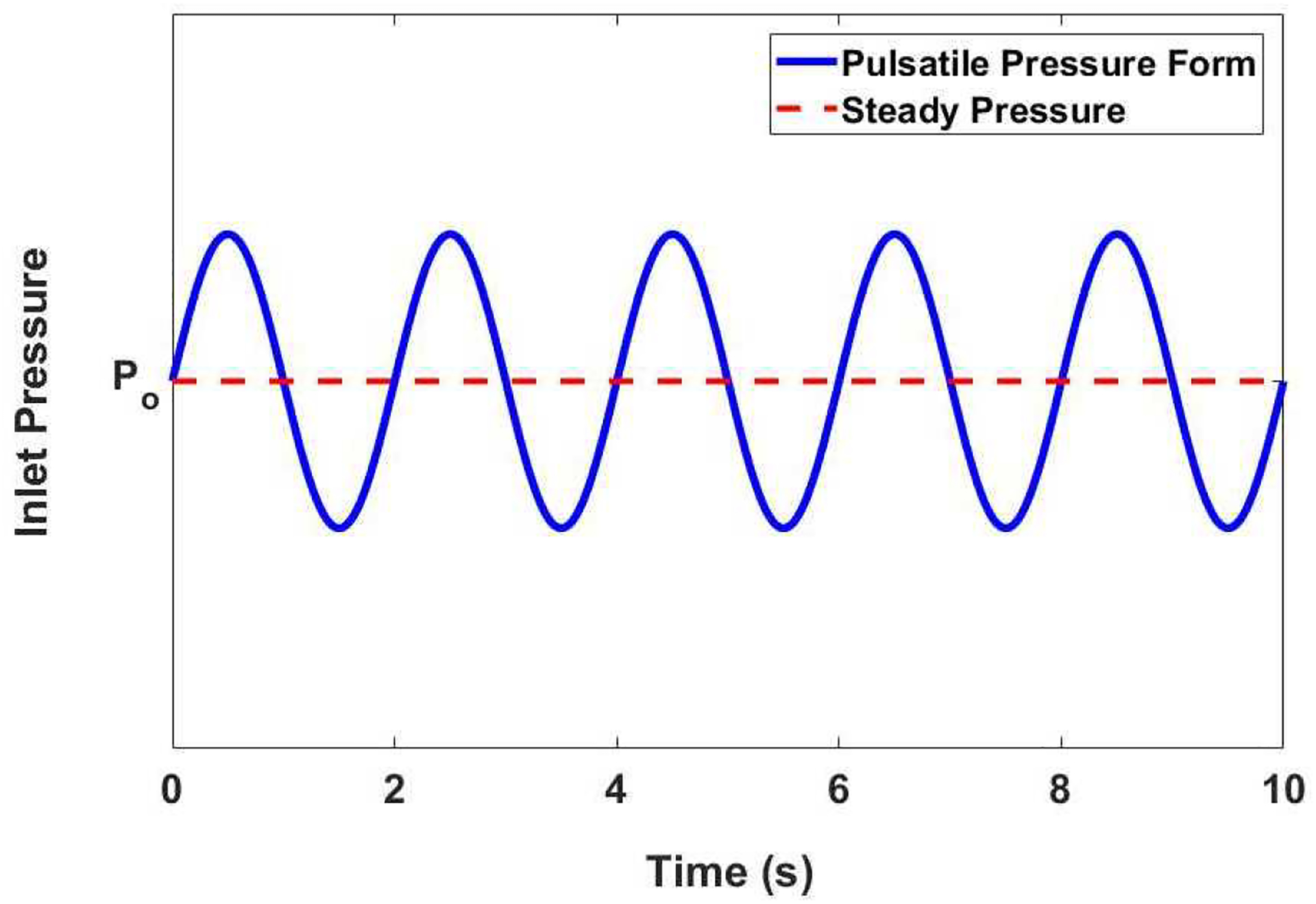
An example pressure boundary condition applied at the inlet, expressed in Eq (2.9). We set our initial condition to be *P*_*o*_ (i.e., the steady pressure component). Here, the angular frequency *ω* = *π rad*/*s* and amplitude *α* = *0.1*.

At the tubule inlet, a pulsatile pressure profile is applied and at the outlet all discharged fluid is considered ‘absorbed’, meaning it exits the simulation volume. Following [39], we express the time-varying component of pressure *p*(*t*) as a sum of steady and oscillatory components. Unfortunately, nomenclature varies within the literature when discussing the steady component (e.g., average, mean, or steady) of pulsatile flow; throughout our analysis, we will define the time-independent pressure component at the inlet as *P*_*o*_ . In addition, we define the oscillatory component as *P*_*θ*_. Before we introduce fully developed pulsatile flow (FDPF), we ramp the inlet pressure until it reaches *P*_*o*_ with a smooth ramp function (continuous second derivative smoothing) defined in COMSOL^®^. When the fluid, solid, and mesh variables (see Numerical Computation) are fully computed, we then introduce the oscillatory component *P*_*θ*_. This allowed us to transition from an equilibrium state to a steady state by introducing FDPF with elastic deformations determined after ramping has completed. We initially prescribed flow velocity, wall displacement, and mesh displacement variables as zero at *t* = *0* since *P*(*t*) = *0*. Furthermore, the inlet pressure *P*(*t*) can be described as

(2.9) $$P(t)=P_{o}+P_{\theta }=\{\begin{array}{ll} P_{o}\psi (t) & t=[0-0.1]\text{ seconds}\\ P_{o}(1+\alpha \text{sin}(\omega t)) & t\geq 0.1\text{ seconds } \end{array}$$

where *ψ*(*t*), *α* and *ω* represents the ramping step function, flow amplitude, and flow angular frequency. Phase *2* (i.e., *t* ≥ *0.1*) in Eq (2.9) is illustrated on Figure 3. Throughout our analysis, we focused on fluid-structure interplay during FDPF. Applying the boundary condition that all discharged fluid is absorbed, we set the outlet pressure boundary condition to zero.

We begin by considering a single driving frequency set equal to the pulse (*ω*/2*π* = *1 Hz* for humans, *ω*/2*π* = *7 Hz* for mice). The assumed boundary condition on fluid elements at the tube wall, the “no-slip” boundary condition ***u***(*r* = *R*_*wall*_)*, requires the fluid to have zero relative velocity at the tube wall. Although detailed measurements to establish or refute the no-slip condition within renal tubules have not been performed, we assume this boundary condition holds. We note that the no-slip boundary condition is violated when transmural flow exists, models of semi-permeable walls require violation of both the no-slip boundary condition and the conservation of mass [7,44]. Additionally, we prescribed weakly coupled displacement along the axial dimension. This constraint is feasible since *in vivo*, tubule axial length is fixed while tubule radius is not. This constraint should be removed when modeling nephrogenesis.

### 2.3. Fluid-structural interactions and parameters

Unlike rigid tubes where fluid velocity can only increase when the driving pressure increases, an elastic tube can radially deform in response to driving pressure (Figure 2), resulting in the exchange of energy between fluid (kinetic energy) and wall (elastic energy). This phenomenon results in qualitatively different flow dynamics as compared to flow within a rigid tube. One difference is the propagation of pressure waves: In a rigid tube, pressure waves propagate with infinite velocity. Another difference is the existence of a non-zero radial fluid velocity component in elastic tubes.

**Figure 4.**
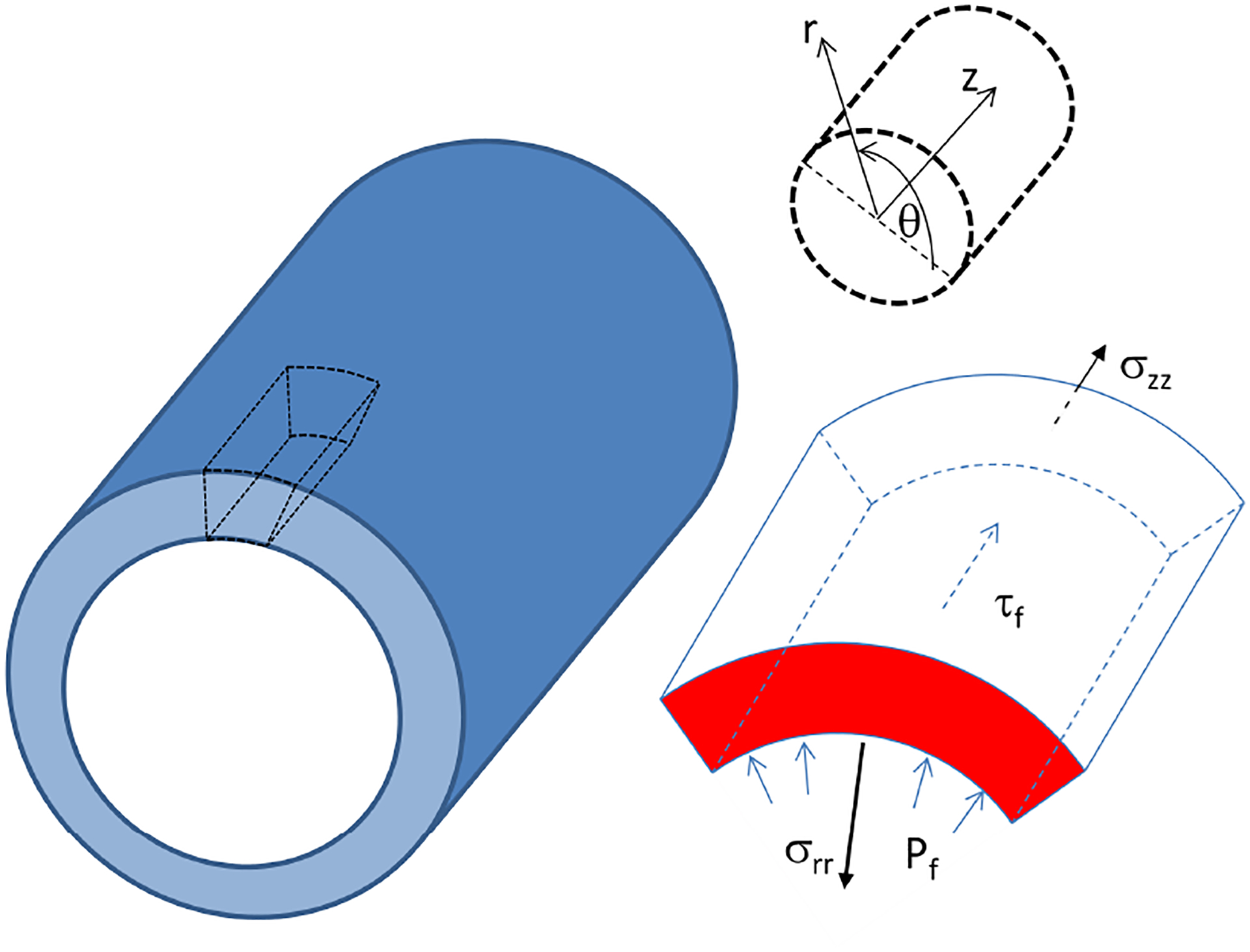
Geometry of force balance between applied fluid pressure *P*_*f*_ as well as shear *τ*_*f*_ , radial *σ*_*rr*_, and axial *σ*_*zz*_ wall stresses.

The stress-strain equations of elasticity govern deformations of the tube wall. In general, the strain has three independent components and the stress tensor has six independent components. In our model, we are most interested in radial strains and axial shear stresses, shown schematically in Figure 4. However, there exists no reported values of the magnitude value of stress found in a singular renal tubule, so we instead focused on analyzing deformation influence on fluid flow and vice versa.

Fortunately, there do exist some well-measured parameters for the system under consideration. In addition to summaries provided in [4], these include: The single-nephron glomerular filtration rate (SNGFR) [45], fluid density and viscosity [46,47], tube diameter and wall compliance [48], and driving pressure dynamics [49]. Values for transmural pressure gradients and permeabilities can be found in [50–52]. Representative values of relevant parameters are provided in Table 1. We do wish to note that of all the parameters on the list, SNGFR may show the most variability over the entire nephron, beginning at approximately *100 nl*/*min* at the entrance and, due to water and solute absorption, drops along the nephron length. The value used here, then, is only applicable to the cortical collecting duct.

## 3. Numerical computation and validation

Here we implement a finite element analysis incorporating the coupled partial differential equations (PDE) system developed in this work in COMSOL^®^ to compute stable solutions. Advantages of this modeling approach include accurate representations of complex geometries, explicit inclusion of material parameters and the ability to capture local effects (e.g., resonant interactions).

Our analysis presented throughout this study involved mesh: *Normal*. This mesh was chosen over other meshes based on results in Table 2 and low computational cost. A total number of *7019* elements with an average element quality *0.866* and an element area ratio of *0.06102*. The mesh area was *1.333* × *10*^−*9*^*m*^*2*^. The mesh contains *477* elements along the edges; in particular, the total number of boundary quadrilateral layers at the interface is *2*. We fixed the maximum and minimum element size to *6.7* × *10*^−*6*^*m* and × *10*^−*8*^*m* with a maximum element growth rate at *1.3*.

During the solver process, we initially ramped the pressure gradient from zero to the steady pressure component (*P*_*o*_). Then as we approached *t* = *0.1*, we added the oscillatory flow component (*P*_*θ*_) to initiate fully developed pulsatile flow. The initial ramping had no influence on the fluid-structure interplay analysis since we analyzed the data between *t* = [*0.1* − *end*]. Furthermore, the initial ramping had no physical influence since we introduced an oscillatory component at the exact time that the pressure gradient reached the steady component value. The advantage of ramping between *t* = *0* − *0.1* was to prescribe initial conditions to zero for the fluid, solid, and mesh variables.

We used the PARDISO solver with Nested dissection multithread for the preordering algorithm. For our approach to solve the FSI problem in COMSOL^®^, we used the fully coupled method which involves simultaneous solution of flow and wall fields. We implemented the Constant (Newton) nonlinear methods with a damping factor of *1*. In addition, our numerical convergence was based on a tolerance termination technique. This technique contained a maximum number of *4* iterations and a tolerance factor of *0.01*. The solvers and methods described above were solved on flow, solid, and moving mesh variables. Lastly, we implemented Matlab R2018b for data post-processing. In this section, computational grid independence and validation of the numerical results will be presented.

### 3.1. Grid study

**Figure 5.**
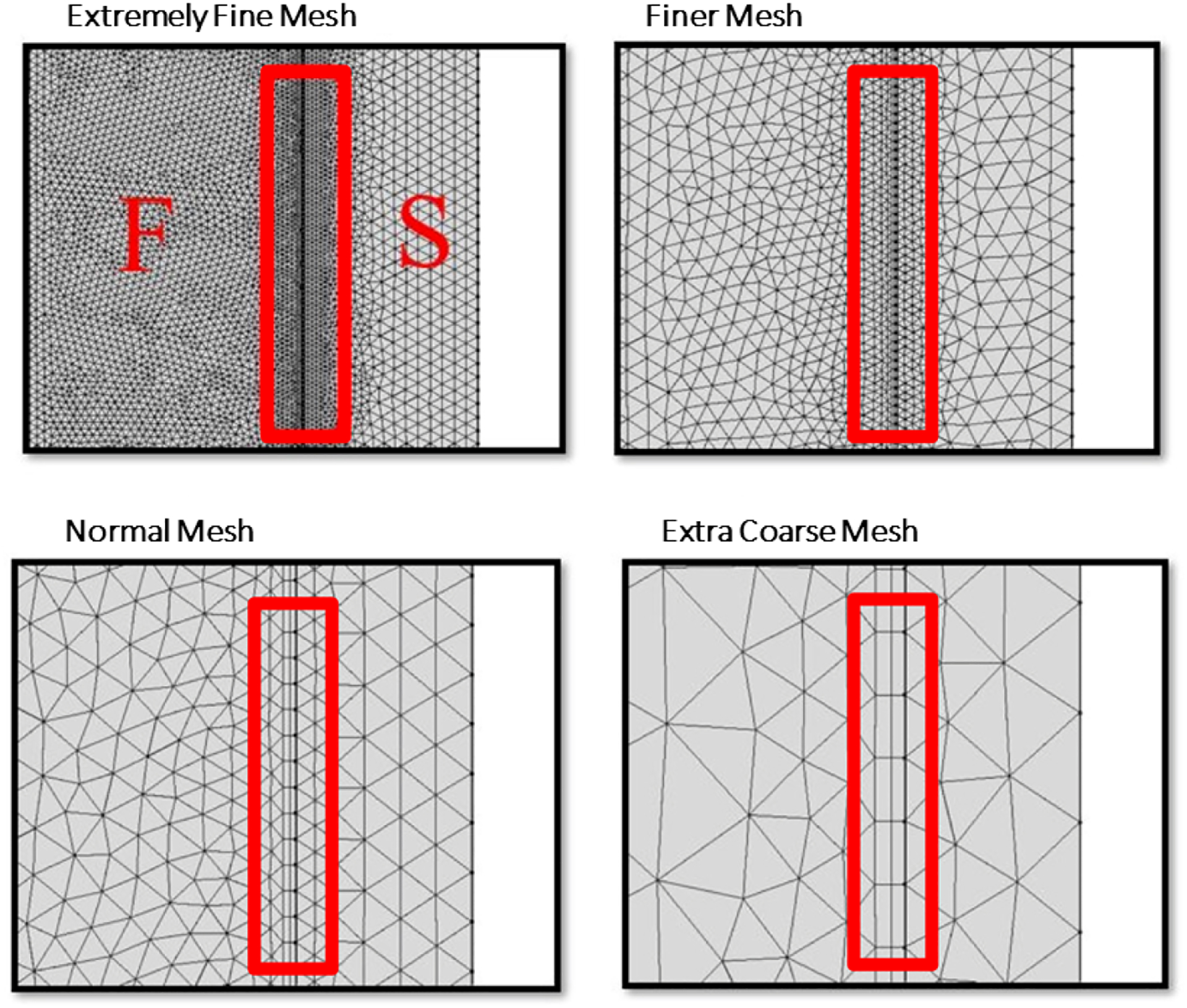
Grid geometry at the interface between the fluid and structural domains, labeled with *F* and *S* respectively for each mesh.

**Figure 6.**
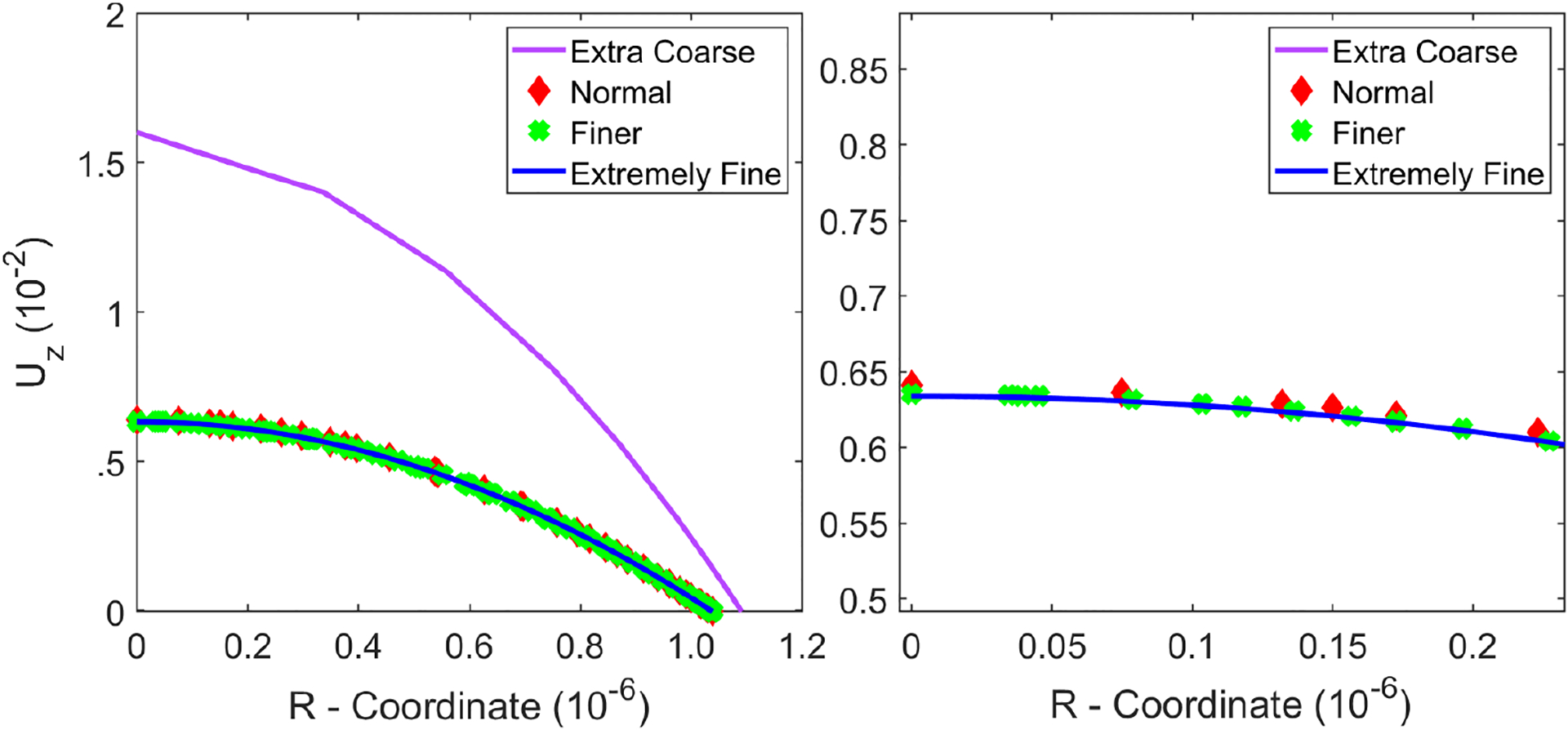
Left: Axial inlet velocity profiles for the four prior mesh geometries at *t* = *1.5s*. Right: A detailed close-up at the center of tube where the fluid velocity is maximum. The peak axial velocity converges to approximately $6.34\frac{\text{mm}}{\text{s}}$ as we refine the mesh.

We studied four different meshes labeled *Extra Coarse*, *Normal*, *Finer*, and *Extremely Fine* in order to analyze the grid independence of the numerical solutions, see Figure 5 and Table 2. These four meshes are labeled in order from most finely discretized grid elements to least finely discretized grid elements. The number of grid elements are listed in Table 2. Figure 5 illustrated the same fluid-structure interface region. The red border enclosed the interacting boundary which is made up of quadrilateral elements rather than traditional triangular elements since quadrilateral elements are preferred for high magnitude loads and displacements. We demonstrated with increasing total number of mesh elements would lead to improved solution accuracy. Importantly for model verification, the solution accuracy eventually converged as we increased total number of meshes elements, scene in Figure 6.

### 3.2. Verification of results

As discussed in the previous section, quadrilateral elements are better suited for deformations [55], so they are generated along the interacting boundary. Shear and normal forces from the fluid act on the wall which deforms the tube structure over time. Triangular elements are used to decrease computational time in areas where there is no fluid-wall interplay, e.g., centered at *r* = *0*. We carry out verification studies focused on both fluid and solid dynamics (e.g., radial velocities, axial velocities, wall stresses and deformations) at different locations and times. This allows for the discovery of possible numerical instabilities as the mesh is refined. However, no divergence or mesh dependency was found.

The results from our studies verify numerical stability by showing improved accuracy for the solutions as the quantity of elements increase, as compared to analytical results when appropriate (e.g., *Hagen-Poiseuille Flow*). In particular, validations were obtained by using idealized base cases (e.g., rigid tube and steady flow) to obtain any evidence of numerical instability, none were seen. No result from structure or fluid outputs showed any solution divergence or mesh dependency during pulsating flow-wall interplay.

## 4. Results

In the present work, the interaction of pulsatile flow is simulated through a single idealized renal tubule located within the renal medulla. Our effort is motivated by a significant difficulty in obtaining *in vivo* fluid flow conditions within a living kidney. Although several experimental approaches have been conducted with varying degrees of success [56, 57], measurements were limited to flow within tubules located near the kidney surface, the renal cortex. Our *in-silico* model provides high-confidence simulations which can be used to study the flow-wall dynamics in the interior kidney, for example the renal medulla. In addition, known perturbed analytical solutions can be retrieved for limiting cases. We present results investigating potential sources of pathology in a manner not possible with current clinical diagnostic modalities.

### 4.1. Fluid-wall interplay and response of the renal tubule

We present results of the effects of pulsatile flow on tubule structure and conversely, the effect of tubule wall compliance on the flow. The relevance of these results is the identification of mechanical properties of the tubule wall and fluid flow (i.e., wall compliance, pulsatile frequency and waveform amplitude) that can lead to testable hypotheses regarding progressive pathologies e.g., *Chronic Kidney Disease* (CKD), *hypertension* and *Polycystic Kidney Disease* in the kidney. These hypotheses will be phrased in terms of progressive changes in wall and flow parameters, as opposed to specific genetic mutations.

**Figure 7.**
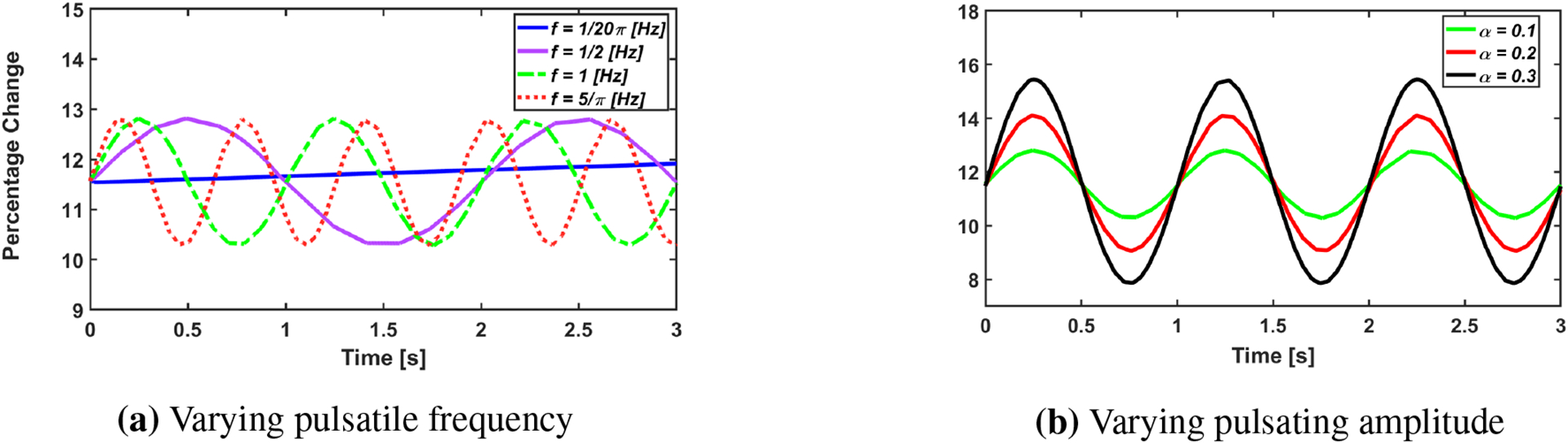
Left: Radial percentage change for four frequencies at fixed *α* = *0.1* and *P*_*o*_ = *13 mmHg*. Right: Analysis of the radial behavior as the amplitudes of the pulsatile flow vary over time at fixed *ω* = *2π rad*/*s*. For both, Young’s Modulus was fixed at *E* = *3* × *10*^*4*^ *Pa*.

In Figure 7, the driving frequency and amplitude were varied and the tubule expansion computed. The radial percentage change was parameterized by $R_{\text{\%}}=1-\frac{\langle R_{\text{wall}}\rangle -R_{\text{wall}}}{R_{\text{wall}}}\times 100$ along the interacting boundary. The notation 〈 〉 represents the average value, for example *R*_*wall*_ is the unstrained tubule radius while 〈*R*_*wall*_〉 represents the average strained radius along the tubule segment. *R*_%_ is a convenient parameter used to investigate how the oscillatory component of flow strains the wall as compared to static flow. In addition, *R*_%_ is an effective parameter describing the accessible fluid volume contained within the tubule via the expression *V*_*fluid*_ = *2πR*_%_*L*.

We present evidence that our model correctly captures pulsatile flow effects on the wall strain in Figure 7a. This figure shows the expansion of the tube segment as a function of *ω* from the flow. Unsurprisingly, we find that low frequencies *ω* ≪ *rad*/*s* approximate steady flow. Our model verifies $\Delta P=\frac{32\mu L\langle u_{z}\rangle }{D^{2}}$, and so in this limiting case, *Hagen-Poiseuille* is an adequate approximation to model renal fluid flow. Δ*P*, *μ*, *L*, 〈*u*_*z*_〉, *D* represent the pressure gradient, fluid viscosity, tube length, averaged axial velocity, and tube diameter. However in most human scenarios, pulsatile angular frequency fluctuations around *α* = *2π*, implying that *Hagen-Poiseuille* is not an adequate approximation for realistic tubular physiology. In addition, we fix *ω* = *π rad*/*s* and computed *R*_%_ as a function of *α* (i.e., the amplitude waveform) in Figure 7b. This figure demonstrates that there is a linear relationship between the pulse amplitude and wall strain.

**Figure 8.**
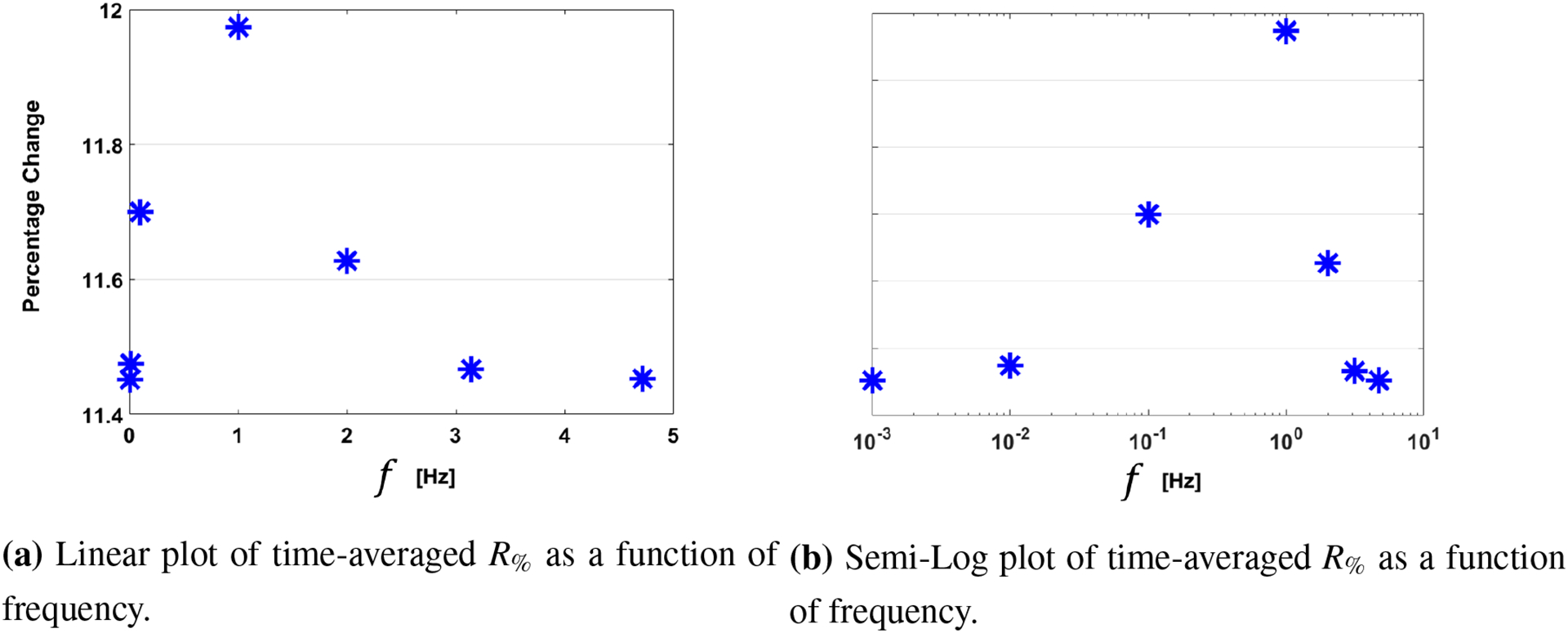
Plots of time-averaged *R*_%_ as a function of frequency. Resonance-type behavior is observed in the neighborhood of *f* = *1 Hz* when assigning *E* = × *10*^*5*^ *Pa*.

We present evidence for resonance-type coupling behavior between flow and wall strain using physiologically relevant parameters in Figure 8. Here, we plot the time-averaged value of *R*_%_ as a function of frequency, both as linear-linear plot and a linear-log plot to more clearly demonstrate that the average radial deformation varies with driving frequency and has a maximum value for a particular driving frequency. Qualitatively, the average radial deformation appears to have the same spectral response as a damped driven oscillator. Interestingly, the peak value of *R*_%_ does not display the same resonance-type behavior (Table 3).

It is important to note that our results were obtained for a single, isolated, tubule. In reality, there is considerable internephron coupling that must be accounted for- both contact-type elastic mechanical coupling and viscous coupling via the interstitial fluid. It has been shown that coupled nephrons and nephron trees can generate irregular oscillations and complex power spectra [58, 59]. Other studies suggest that internephron coupling may prompt synchronization, quasiperiodicty, and perhaps chaos in a nephron tree [60, 61]. Internephron coupling is beyond the scope of this report, which is simply focused on creating a validated model. Our model can be expanded to incorporate hydro-elasto-mechanical internephron coupling and that is one goal of future modeling efforts.

Regardless, our results demonstrate a method to explore how varying flow parameters (e.g., amplitude and frequency) can significantly alter the strain dynamics of nephrons. Our model shows that there is a relationship between flow waveform and wall strain. Moreover, this knowledge can provide insight into understanding more complex geometries, for example that found in nephron trees.

### 4.2. Effects of wall compliance on renal tubular fluid flow

To evaluate the effects of wall compliance on the dynamical variables related to pulsatile flow of incompressible Newtonian fluid inside the elastic renal tubule, the wall elastic modulus was varied (e.g., *E*= *3* × *10*^*4*^, *1* × *10*^*5*^, *1* × *10*^*8*^ *Pa*) to model physiologically realistic tubule wall properties [54]. The importance of this parameter study is to investigate, for example, tubular atrophy or tubular fibrosis. Tubular atrophy is a general term that describes tissue phenotypes due to chronic tubular injuries, resulting is the tubular basement membrane becoming increasingly stiff. Altered wall stiffness also occurs in other physiological systems, notably the vasculature (arteriosclerosis), pulmonary (cystic fibrosis) and liver (biliary fibrosis).

**Figure 9.**
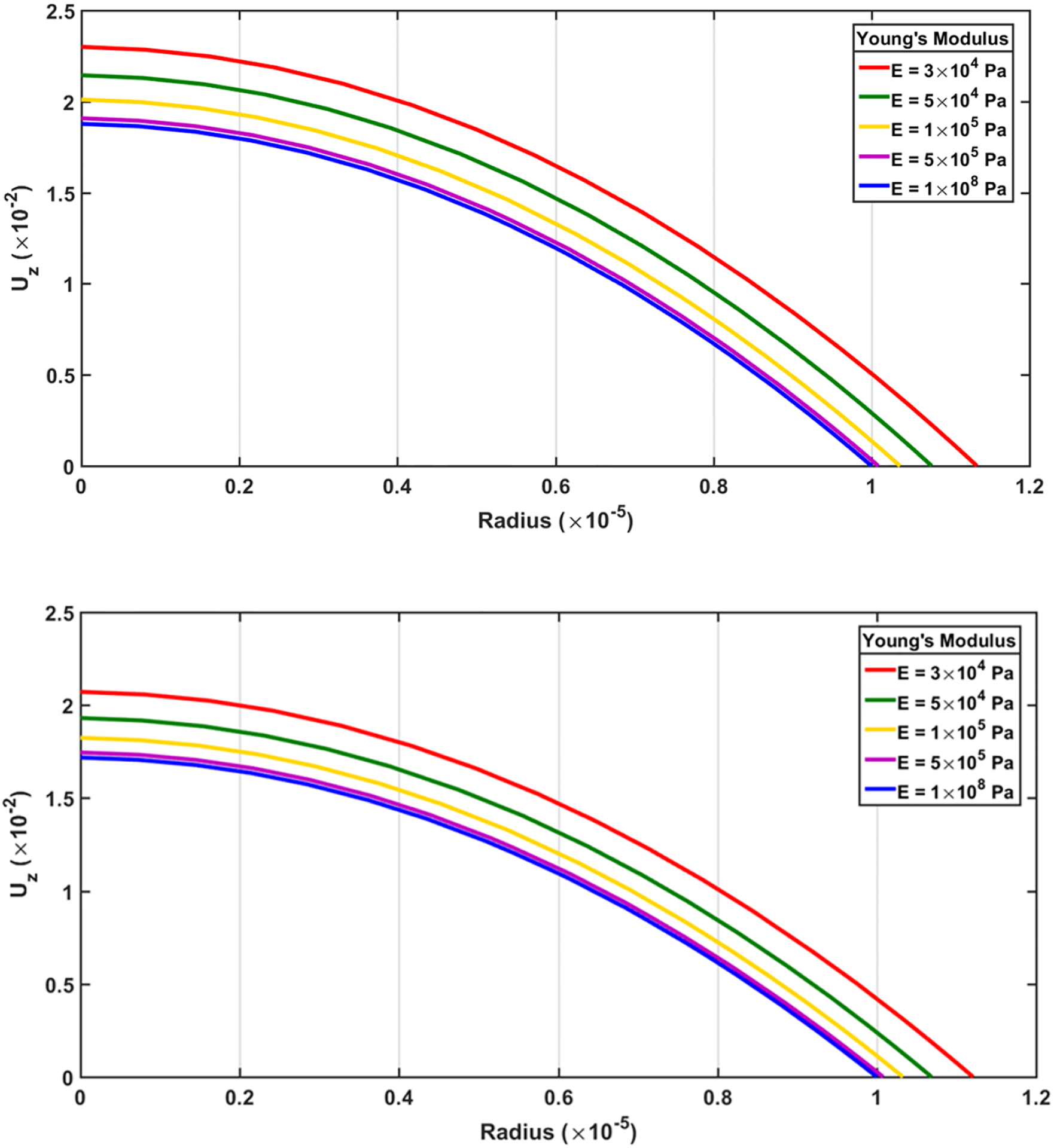
Top and bottom velocity profiles present the corresponding time (*t* = *1.25*, *1.5 s*). Peak *u*_*z*_ and radial expansion can be seen to differ with compliance.

**Figure 10.**
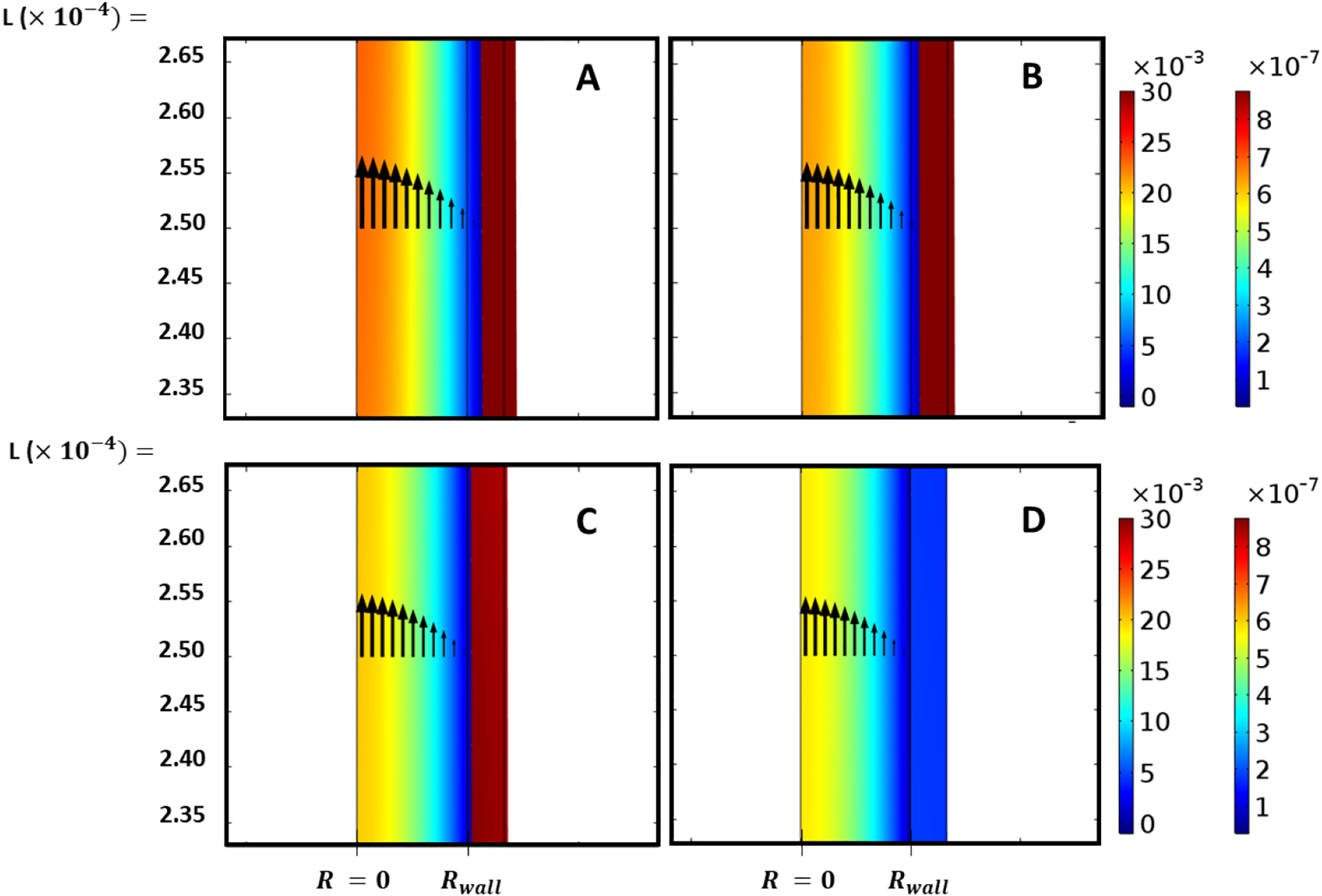
Animation based on Figure 9 at time (*t* = *1.25 s*) representing fluid-wall interplay. A, B, C and D present the corresponding Young’s Modulus (*E* = *3* × *10*^*4*^, *5* × *10*^*4*^, *1* × *10*^*5*^, *5* × *10*^*5*^ *Pa*). Total velocity and radial expansion can be seen to differ with compliance.

A numerical study was conducted by varying the compliance of the wall structure when all other boundary conditions and properties of the fluid and tubule wall remained unchanged. As can be seen in Figure 9, as the elastic modulus increases from *3* × *10*^*4*^ to *1* × *10*^*8*^ *Pa*, the maximum values of axial velocity decreases. Furthermore, in the limiting case *E* ≫ *3* × *10*^*4*^ *Pa* the wall acts as a rigid tubule since radial expansion is negligible. By increasing the elastic modulus, the rigidity of walls increases and the radial deformation decreases; the radial displacement is shown in Figures 9 and 10.

**Figure 11.**
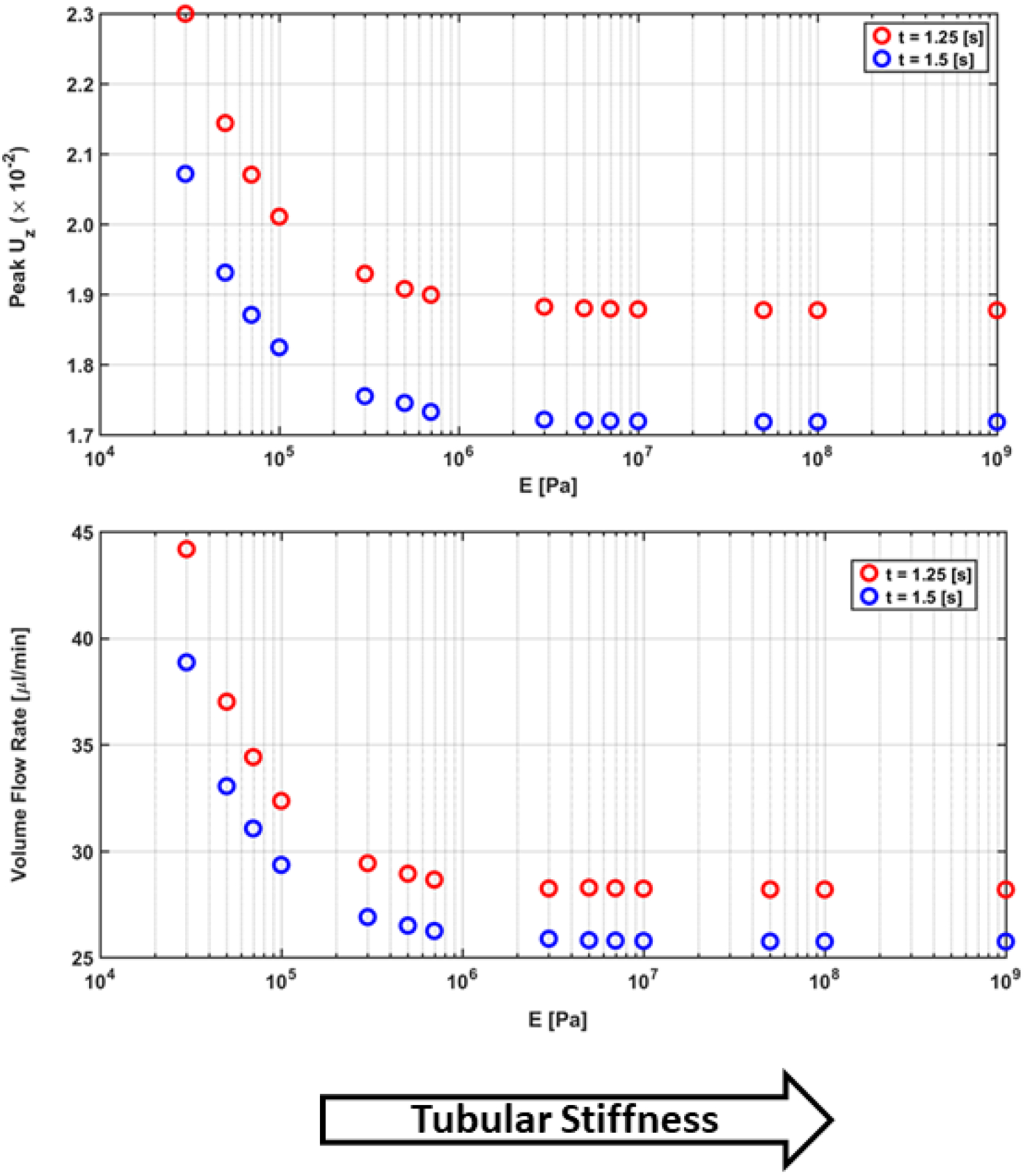
Plot of the peak *u*_*z*_ and *Q* at the midline of the tubular structure during time *t* = *1.25*, *1.5s* as tubular stiffening occurs. Axial velocity and volume flow rate are in units of centimeters per second and microliters per minute. Parameters *α*, *P*_*o*_, *ω* was fixed at *0.1*, *13 mmHg*, and $2\pi \frac{\text{rads}}{s}$.

In Figure 11 and Table 4, the peak axial velocity and volume flow rate dramatically decrease as flexibility of tubular wall decreases and reaches an asymptotic value. This asymptote represents the limiting case - rigid walls. As seen in the figures, this demonstrates an important interplay between wall stiffness and fluid transport rates.

In addition, as the elastic stiffness of the renal tubules decreases, the kidney requires more power to pump renal fluid through the nephron. Hence, the system could experience structural degeneration and increased system pressure (e.g., hypertension). Similarly, a reduction of renal tubule elasticity leads to the development of Chronic Kidney Disease. Therefore, the efficiency of kidney function decreases with decreasing elasticity which supports the notion of dynamic pathology.

## 5. Summary and conclusions

In this paper, kidney flow biomechanical environments corresponding to pathophysiological regimes were investigated by examining fluid-structure interactions and solving partial differential equations in Arbitrary Lagrangian-Eulerian formalism using finite element modeling techniques. In addition, analysis of fluid and structure parameter studies as a model for several pathologies was performed. Although we present an imperative approach within investigating renal dynamics, our numerical simulations are limited to modeling the distal nephron. Within future studies, we plan to model entire nephron dynamics by incorporating unsteady pressure perturbations, reabsorption and releasing the axisymmetric geometry assumption. Our conclusions are summarized as:

1. Our model shows that a better understanding of driving pressure waveforms (both frequencies and amplitudes) involved deep within the medulla is needed as elastohydrodynamic coupling between neighboring nephrons could strongly modify fluid throughput.
2. In the case of driving pressure frequency influence on distal tubular wall dynamics, our model shows support in approximating renal flow as *Hagen-Posiuille* in the limiting case *ω* ≪ *π*. Our model demonstrates that there exists a *ω* that maximizes radial expansion during local time scales.
3. From the results of flow in stiffening distal convoluted tubules, the peak axial velocity monotonically decreases as the compliance decreases. Our investigation provides important links between biomechanical environments and possible kidney function failures, demonstrating that fluid volume flow rates decrease as the structural parameters of the wall transition to pathological values.
4. Our model provides the ability to analyze local tissue stresses in damaged or degenerated nephrons. For many chronic diseases, tissue damage accumulates over long periods of time; our model allows us to improve understanding of the progressive damage due to hypertension and diabetes.
5. Since detailed knowledge about the actual renal fluid flows experienced by cilia in a living kidney is largely lacking, this research effort provides important information regarding design [4] of perfused tissue and organ experiments.

## Figures and Tables

**Table 1.** Model system parameter values.

Parameter name	Symbol	Representative value	Reference(s)
Inner radius of tube	*R*_*wall*_	*1.0 × 10*^*1*^ *μm*	[48]
Wall thickness	*h*	*1,0 × 10*^*1*^ *μm*	[48]
Young’s Modulus of tube wall	*E*_*wall*_	*1.0 × 10* ^*5*^ *Pa*	[54]
Poisson ratio of tube wall	*V*_*wall*_	*3.0 × 10* ^*−1*^	
Density of wall material	*ρ*_*wall*_	*$1.1\;\times \;10^{3}\frac{\text{kg}}{m^{3}}$*	[53]
Density of fluid	*ρ*_*fluid*_	*$1.01\;\times \;10^{3}\frac{\text{kg}}{m^{3}}$*	[46]
Dynamic viscosity of fluid	*μ*_*fluid*_	*$5.0\;\times \;10^{-3}\frac{\text{Pa}}{s}$*	[47]
SNGFR	*Q*	*$5\frac{nl}{\text{min}}$*	[45]
Time-averaged pressure	*P*_*o*_	*1.0 × 10*^*−1*^ *kPa*	[49]
Length of tube segment	*L*	*1.0 cm*	[54]
Shear stress exerted on wall	*τ*_*w*_	*to be calculated*	N/A

**Table 2.** Mesh geometries.

Mesh	Triangles	Quads	Vertices	Solution Time	Peak(*u*_*z*_) *[cm/s]*
Extra Coarse	*1146*	*174*	*799*	*1 mins. 59 secs.*	*16.0185*
Normal	*6591*	*428*	*3856*	*7 mins. 10 secs.*	*6.4112*
Finer	*28,172*	*1156*	*15503*	*28 mins. 00 secs.*	*6.3554*
Extremely Fine	*137,391*	*2, 240*	*71,505*	*2 hrs. 11 mins. 15 secs.*	*6.3410*

**Table 3.** Numerical results of Figure 7.

*f Hz* at *α = 0.1*	mean(*R*_%_)	max(*R*_%_)
0.001	11.45	11.47
0.01	11.48	11.50
0.1	11.70	11.94
1	11.97	12.71
2	11.46	12.69
π	11.42	12.71
1.5π	11.29	12.71

**Table 4.** Numerical results of Figure 9.

$E\frac{N}{m^{2}}$	$\text{peak}\left(u_{z}(t=1.25s)\right)\frac{cm}{s}$	$\text{peak}\left(u_{z}(t=1.5s)\right)\frac{cm}{s}$	$Q(t=1.25)\frac{\mu l}{min}$	$Q(t=1.5)\frac{\mu l}{\text{min}}$
3 × 10^4^	2.2996	2.0716	44.1770	38.8567
5 × 10^4^	2.1439	1.9310	37.0092	33.0431
7 × 10^4^	2.0704	1.8707	34.4082	31.0496
1 × 10^5^	2.0105	1.8244	32.3964	29.3401
3 × 10^5^	1.9293	1.7549	29.4193	26.8891
5 × 10^5^	1.9077	1.7451	28.9257	26.4890
7 × 10^5^	1.8993	1.7325	28.6464	26.2376
3 × 10^6^	1.8822	1.7214	28.2334	25.8702
5 × 10^6^	1.8801	1.7200	28.2701	25.8012
7 × 10^6^	1.8792	1.7195	28.2450	25.7830
1 × 10^7^	1.8786	1.7191	28.2246	25.7695
5 × 10^7^	1.8775	1.7183	28.1887	25.7439
1 × 10^8^	1.8774	1.7182	28.1845	25.7406
1 × 10^9^	1.8772	1.7181	28.1809	25.7376

## Footnotes

* *R*_*wall*_ is a fixed radius during static flow; however, the tubular wall will experience deformations leading to many other possible zero relative velocities.
